# A new, fluorescence-based method for visualizing the pseudopupil and assessing optical acuity in the dark compound eyes of honeybees and other insects

**DOI:** 10.1038/s41598-021-00407-2

**Published:** 2021-10-28

**Authors:** Elisa Rigosi, Eric J. Warrant, David C. O’Carroll

**Affiliations:** Department of Biology, Lund University, Sölvegatan 35, 22362 Lund, Sweden

**Keywords:** Retina, Animal physiology, Zoology

## Abstract

Recent interest in applying novel imaging techniques to infer optical resolution in compound eyes underscores the difficulty of obtaining direct measures of acuity. A widely used technique exploits the principal pseudopupil, a dark spot on the eye surface representing the ommatidial gaze direction and the number of detector units (ommatidia) viewing that gaze direction. However, dark-pigmented eyes, like those of honeybees, lack a visible pseudopupil. Attempts over almost a century to estimate optical acuity in this species are still debated. Here, we developed a method to visualize a stable, reliable pseudopupil by staining the photoreceptors with fluorescent dyes. We validated this method in several species and found it to outperform the dark pseudopupil for this purpose, even in pale eyes, allowing more precise location of the gaze centre. We then applied this method to estimate the sampling resolution in the frontal part of the eye of the honeybee forager. We found a broad frontal acute zone with interommatidial angles below 2° and a minimum interommatidial angle of 1.3°, a broader, sharper frontal acute zone than previously reported. Our study provides a new method to directly measure the sampling resolution in most compound eyes of living animals.

## Introduction

### (a) Quantification of visual acuity in compound eyes

The compound eyes of arthropods are comprised of densely packed tube-like optical units known as ommatidia, each with a lens that focuses light from a narrow region of visual space onto underlying photoreceptors. Together, the ommatidia sample the region of space viewed by each eye and allow animals to resolve information in different locations of their environment. At a single ommatidium level, optical resolution of the most common type of compound eye, the apposition eye, can be well predicted from measured or inferred parameters such as photoreceptor diameter, focal length and facet diameter^1–3^. However, even the best quality image is only useful if it is matched by an angular sampling strategy to appropriately exploit the potential resolution^1–3^. The independent sampling of space by individual ommatidia in apposition eyes provides a large number of free parameters for local variations in sampling strategy. The diameter of individual facet lenses, as well as the angular density of the ommatidia, and how they are geometrically juxtaposed, all change radically among species, sexes and across eye regions within the same compound eye^1,4^. These characteristics determine differences in the ability of the eye (or a local region of an eye) to sample the environment and thus to resolve different objects.

Over the last century many different methods have been used to measure or infer the sampling resolution of compound eyes. In living animals, indirect measures have exploited the behavioural responses to variously fine spatial patterns in either freely moving (e.g.^5–10^) or tethered insects (e.g.^11–13^) as well as direct electrophysiological measures from the first optic ganglion^14,15^. Estimates have also been obtained from a number of different methods applied to fixed (non-living)^16–19^ and living^20^ animals to estimate the gaze direction of individual ommatidia based on the anatomy of the eye.

Each of these techniques, however, have inevitable pitfalls. Behavioural estimates, for example, are easily confounded by the motivation of the animal to solve a difficult task. Electrophysiological measures are technically very challenging and often neglect differences in resolution across the eye in favour of recording from the most easily accessed regions. Anatomical measures based on samples preserved in fixative are limited by the non-physiological state of the eye as well as artefacts such as shrinkage due to chemical treatments, which may be severe even with optimal fixation and embedding^21^. For many years the ‘pseudopupil method’ has provided a more direct approach for measuring the axial directions of ommatidia in living insects. This method most often relies on the principal pseudopupil, a dark spot which results from light absorption by the pigment cells surrounding the crystalline cone and the photoreceptors close to the direction of gaze^22^. This dark pseudopupil shifts its position as the eye rotates, and changes shape according to the density of receptors sampling the local viewing direction. Mapping the pseudopupil’s positional shift for a known rotational angle allows estimation of the sampling resolution, as measured by the divergence angle between neighbouring ommatidia (known as the interommatidial angle, Δ*φ*). This angle describes the local ommatidial packing density, that is, the number of ommatidia that view a specific narrow region of visual space. However, attempts to estimate the visual acuity in this way has, in many insects, been frustrated by the lack of a clearly identifiable principal pseudopupil due to pigments in pigment cells underneath the cornea that make the eyes appear uniformly dark.

### (b) Visual acuity in honeybees

In this context, the honeybee, an important model in neuroethology and cognition^23^, has for almost a century been the focus of debate concerning the sampling resolution of its darkly pigmented eyes and many attempts have been made to measure it, with inconsistent results (^15^; also reviewed in^24^). This exemplifies the implicit difficulties of obtaining reliable measures of optical acuity in darkly pigmented insect eyes. In the honeybee (as in many Hymenoptera) this is further complicated by the vertically elongated, oval-shaped eye, resulting from different sampling in the vertical and horizontal directions, leading to some confusion in defining the interommatidial angle along these dimensions which differs among authors^22^.

The first attempts to measure the sampling resolution of the honeybee eye date back to 1928, when Baumgärtner applied anatomical reconstruction of the ommatidial axes based on histological sections^16^. He found a resolution maximum close to the equator of the eye and 60° away from the frontal visual field, with a vertical resolution, Δ*φ*_*v*_, just below 1°, and with a horizontal row separation, Δ*φ*_*h*_, of around 2.6°^16,25^. These numbers, however, require allowance for the hexagonal nature of the ommatidial lattice, where Δ*φ* = [Δ*φ*_*h*_^*2*^ + Δ*φ*_*v*_^*2*^]^0.5^ (for a detailed overview of this formula, see^22^). Once this allowance has been made a Δ*φ* of 1.64° can be calculated*.* Around the same time, Hecht and Wolf^5^ studied the reaction of crawling bees to grating patterns of different stripe widths and found a resolution agreeing with Baumgärtner’s anatomically-derived value. A similar value for the vertical resolution, around 1°, was then shown in a later study by Del Portillo^26^ by reconstructing the surface of the cornea from anatomical sections. In 1971 Wiitanen and Varela^27^ estimated Δ*φ* from histological sections and obtained a minimum of 1.5°, averaged from the vertical and horizontal components. In subsequent work, a larger value of 2° was supported by analysis of freely-flying honeybees trained with sugar rewards in a Y maze: by varying the density of gratings Srinivasan and Lehrer^6^ found a resolution limit for gratings with bars of 2° width, a value also confirmed by a later study in walking bees^28^.

In the honeybee, the more direct pseudopupil method is challenged by screening pigment in the eye. In 1973 Kirschfeld^29^ used antidromic illumination and measured the radiation of ommatidial axes at different focal planes above the cornea to estimate an average Δ*φ* of 1.8° in the frontal eye*.* Unpublished PhD dissertation work by Seidl^30^ also used antidromic illumination and estimated a minimum Δ*φ* of 2.1° horizontally and 1.2° vertically in the middle-frontal part of the eye. However later work by Horridge^24^ claimed that Seidl’s method included errors in interpretation of the data such that the minimum Δ*φ* were actually isotropic at around 1.7°, and hence may agree more closely with Kirschfeld’s estimate for frontal acuity^29^.

To add to this confusion, a recent paper^18^ exploited X-ray micro computed tomography techniques (µCT) to investigate the visual properties of the honeybee eye, obtaining a minimum inter-facet angle (a measure that approximates the average Δ*φ* along all 3 axes of the ommatidial rows) of about 1°, but not at the front of the eye—instead at an elevation of 30° and an azimuth of 60°. This conflicts with our own intracellular electrophysiological measures of resolution in honeybee photoreceptors^31^, which suggested that acuity is highest directly at the front of the field of view.

The disparity between these various attempts in both the value and location of highest visual acuity underscores the need for a more reliable and accurate method to measure Δ*φ* across a living eye, particularly in dark-pigmented eyes like those of the honeybee. To solve these difficulties and controversies we here describe a new method, whereby uptake of fluorophores by the photoreceptors induces a fluorescent pseudopupil—even in darkly pigmented insect eyes. We applied this technique to several insect species, including some in which we could also observe a dark principal corneal pseudopupil when viewed with incident illumination. We hereafter refer to this as the ‘dark’ pseudopupil to differentiate it from our bright induced fluorescent pseudopupil and from a variety of other pseudopupil types that have previously been observed in different insect species (see Methods section). This allowed us to confirm that the induced fluorescent pseudopupil provides a reliable estimate for the gaze direction of ommatidia in living (intact) animals. We then applied this technique to accurately map Δ*φ* in the frontal visual fields of honeybee foragers. Our data confirm the presence of an intense frontal acute zone in these insects, with Δ*φ* reaching a minimum of 1.3° and remaining below 2° across a large region of the frontal eye (e.g. traversing 40° of azimuth and 60° of elevation). Taken together with our own recent electrophysiological estimates of photoreceptor acceptance angles down to 1.6° within this frontal acute zone, we confirm that the frontal resolution of honeybee forager eyes is similar to that found in many dipteran flies.

## Results

### (a) Induction of the fluorescent pseudopupil

**Figure 1:**
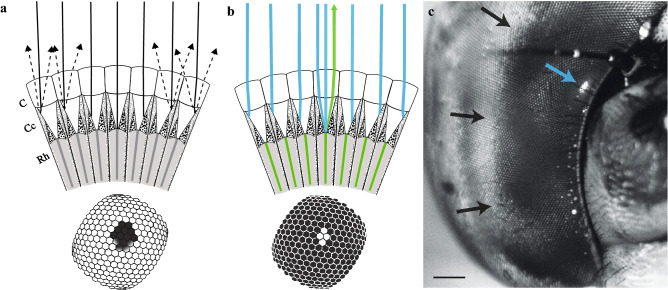
Dark and induced fluorescent pseudopupils. **(a)** Section of an insect apposition compound eye illustrating formation of the principal pseudopupil when illuminated by incident co-axial light (*continuous black lines*); modified after^22^. To the experimenter, looking through the lens of the microscope, the surface of the eye (*diagram below*) will appear pale in the majority of the facets where incident light is scattered from secondary pigment cells (*black dashed lines*). However, in ommatidia aligned with the viewing axis, light is absorbed by rhabdoms and primary pigment cells, making them appear dark (*diagram below*). *C* corneal facet, *Cc* crystalline cone, *Rh* rhabdom. **(b)** Section of an apposition compound eye illustrating formation of the fluorescent pseudopupil. In this case the compound eye has been injected with a fluorescent dye that has been taken up by the rhabdomeres (*green shade* in the diagram, e.g. Lucifer Yellow or Neurobiotin 488, see “Methods” section and Fig. 2). Short wavelength incident light (*blue shade*) will excite the fluorophore in the rhabdomeres so that only those ommatidia aligned with the optical axis of the microscope will re-emit longer wavelengthlight (*green line*). These re-emitted light rays will cause the facet lenses through which they pass to be bright compared to the surrounding facet lenses which will be dark (*diagram below*). **(c)** Images taken from the eye of a dragonfly, *Aeshna cyanea*, whose induced fluorescent pseudopupil was obtained using Lucifer Yellow (see “Methods”). Scale bar: 500 µm. Images from Supplementary Information video 1, part 1. *A. cyanea* has a frontal acute zone that is very broad (*black arrows*) as seen by the dark pseudopupil occupying a large area of the frontal part of the eye when the animal is perpendicular to the axis of the objective, i.e. at 0° longitude. However, the induced fluorescent pseudopupil (*blue arrow*) is easily identified and because it is confined to a smaller region of the eye, its centre is easy to pinpoint.

Dark principal pseudopupils result from absorption of orthodromic light by the rhabdoms and surrounding primary pigment cells^22^. This gives rise to the appearance of a dark spot centred on the photoreceptors directed towards the observer (Fig. 1a). This becomes visible by contrast with light substantially scattered by secondary pigment cells just below the cornea^22^. To visualise the fluorescent pseudopupil, application of the combination of dimethylsulfoxide (DMSO), ringer solution and a fluorescent neuronal tracer (either Neurobiotin 488 or Lucifer Yellow) appears to retrogradely label the photoreceptors (or at least their rhabdomeres) in the living eye. Short-wavelength light focused by the insect’s own cornea onto the cells below, then presumably excites longer wavelength fluorescence within any structures that take up the dye, and at least some of this light then propagates back along the incident path (Fig. 1b). This can then be selectively visualized using an appropriate fluorescence filter set (see Methods). Figure 1c illustrates an example of such a fluorescent pseudopupil induced in a dragonfly, *Aeshna cyanea*. By rotating the eye, this induced fluorescent pseudopupil clearly moves across the eye surface (see Supplementary Information video 1, part 1), also changing in its shape and dimensions in response to variations in the local photoreceptor (i.e. ommatidial) angular density, just as seen in the natural dark pseudopupil^22^.

Does the signal we detect as the fluorescent pseudopupil truly originate from the photoreceptors, or could it be due to uptake of the fluorophore by other cells within the ommatidium? This is an important issue to resolve if we are to trust that the centre of this pseudopupil defines the true gaze direction of the ommatidia and can thus be used as alternative to the dark (principal) pseudopupil to map visual acuity in species where the latter is invisible.

**Figure 2:**
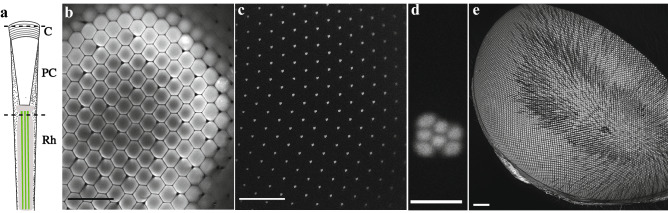
The fluorescence of the induced fluorescent pseudopupil originates from the rhabdomeres. **(a)** Diagram of a single ommatidium of a dipteran compound eye. Dotted lines denote the plane where optical cross sections of the eye in (b–d) were taken. From top to bottom: corneal facet (C), pseudocone (PC) and distal tip of the rhabdomeres (Rh). **(b.c)** *Eristalis tenax* compound eye after application of Lucifer Yellow and scanned with a confocal microscope, with a 63× glycerol objective. Scale bar 100 µm. The fluorescence observed at the surface of the eye **(b)** originates from the fluorescent rhabdomere tips as no other cells or parts of the photoreceptors are fluorescent when we focus below the cornea to the plane of the rhabdom tips **(c)**. **(d)** Magnified view from a single ommatidium, showing the distinctive trapezoidal shape formed by the distal tips of 7 adjacent rhabdomeres, typical of dipteran flies. Scale bar 5 µm. **(e)** Maximum intensity projection of a z-stack of the left eye of a female *Eristalis tenax*. When the dye (in this case Neurobiotin 488) had been left in the head for more than 3 h we experienced glowing in the entire eye. Image acquired with a Leica SP8 DLS confocal microscope and a 2.5× air objective lens (see also Supplementary Information video 1, part 2).

The eyes of dipteran flies are ideal for testing this hypothesis, since Franceschini^32^ previously showed via corneal neutralization that the majority of the refracting power in fly ommatidia is due to curvature of the outer corneal facets. The distinctive trapezoid pattern of the distal tips of the rhabdomeres in each dipteran ommatidium can then be imaged using a conventional microscope by simply neutralizing the refracting power of the cornea with a drop of high-refractive index oil and illuminating the retina from behind with light introduced through an aperture cut into the back of the head, i.e. antidromic illumination^32^. We adapted this approach to epifluorescence illumination in a scanning laser confocal microscope. We first applied the same method as in Fig. 1 to induce a fluorescent pseudopupil in the clear eye of the dipteran hoverfly, *Eristalis tenax* (Fig. 2a–d, about 20 min after application of the dye). We then applied a drop of glycerol to both sides of a glass coverslip placed in contact with the eye to neutralise the corneal refraction. This allowed us to use a long working distance, high numerical aperture glycerol immersion objective to obtain high resolution optical sections from different focal planes, from the cornea to the distal parts of the fluorescing photoreceptors (Fig. 2a–d). At high magnification the typical dipteran trapezoidal pattern was then clearly visible against a completely dark background (Fig. 2c). This shows that it is primarily the rhabdomeres, rather than other organelles or cells within each ommatidium, that initially take up large amounts of the fluorophore. It is worth noting, however, that in the hoverfly the fluorescence subsequently spreads to a large number of supporting cells just beneath the cornea. After around three hours, the induced fluorescent pseudopupil may be swamped by fluorescence from the entire corneal surface of the eye as viewed from any angle (Fig. 2e, and Supplementary Information video 1, part 2).

### (b) Fluorescence allows for a better estimate of the pseudopupil centre

**Figure 3:**
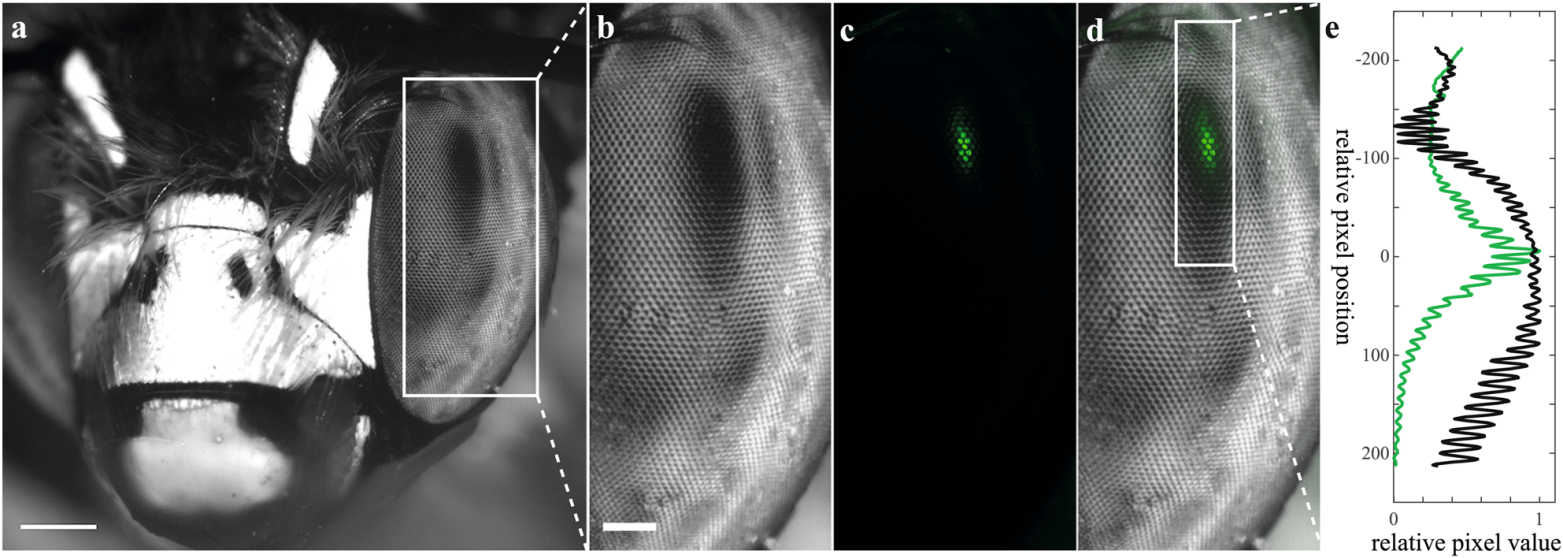
Induced fluorescent and dark pseudopupil overlap. **(a)** Head of the bee *Anthophora *sp. These bees have clear eyes and its dark pseudopupil is evident as a black spot on the eye surface. Image acquired with a Nikon SMZ18 equipped with a bright field filter cube (P2-EFLBF) and a ¼ wave plate. The eye was stained with Neurobiotin 488 (see “Methods”). Scale bar: 500 µm. **(b)** Zoomed-in view of the area marked by the *white rectangle* in **(a)** showing more clearly the dark pseudopupil in the left eye. It is common to see multiple pseudopupils resulting from the incident light from the environment around the eye. **(c)** Zoomed-in view of the area marked by the *white rectangle* in **(a)**, after using a P2-EFLGFP-B filter cube—the induced fluorescent pseudopupil was visible in the same sample (in vivo). **(d)** Co-visualization of the dark **(b)** and induced fluorescent pseudopupil (c) showing a clear overlap of the two. **(b–d)** Scale bar: 200 µm. **(e)** Relative pixel values for the induced fluorescent and dark pseudopupils in the area marked in **(d)** by a *white rectangle*. Note that the centres of the two (i.e. max relative pixel value) coincide, with the induced fluorescent pseudopupil having the centre much more easily identifiable compared to the dark pseudopupil.

Can the induced fluorescent pseudopupil reliably locate the direction of gaze as successfully as the dark pseudopupil observed in some compound eyes? Given that we know from the evidence presented above that the dye is selectively taken up by the rhabdomeres, we would predict that only light focused directly onto their tips should excite fluorescence. The resulting pseudopupil should then be confined to the centre of a region spanned by the dark pseudopupil (which is broader because light is also absorbed by the surrounding pigment granules from off-axis rays). To test this, we stained the photoreceptors of a solitary bee, *Anthophora *sp., in which the majority of the corneal surface appears pale due to strongly scattering secondary pigment so that a well contrasted dark pseudopupil can easily be seen from many viewing angles (Fig. 3a,b). We captured sequential images using first orthoscopic illumination with white light to visualise the dark principal pseudopupil (Fig. 3b) and then epifluorescence illumination to detect the fluorescent pseudopupil (Fig. 3c) so that we could then construct a false-colour montage (Fig. 3d). This demonstrates that the fluorescent pseudopupil is coincident with the darkest part of the principal pseudopupil. We quantified this further by constructing the intensity profiles across the pseudopupil centres (Fig. 3e). This shows that the two share a common centre but for the fluorescence image also reveals a sharper fall-off in brightness away from the pupil centre.

**Figure 4:**
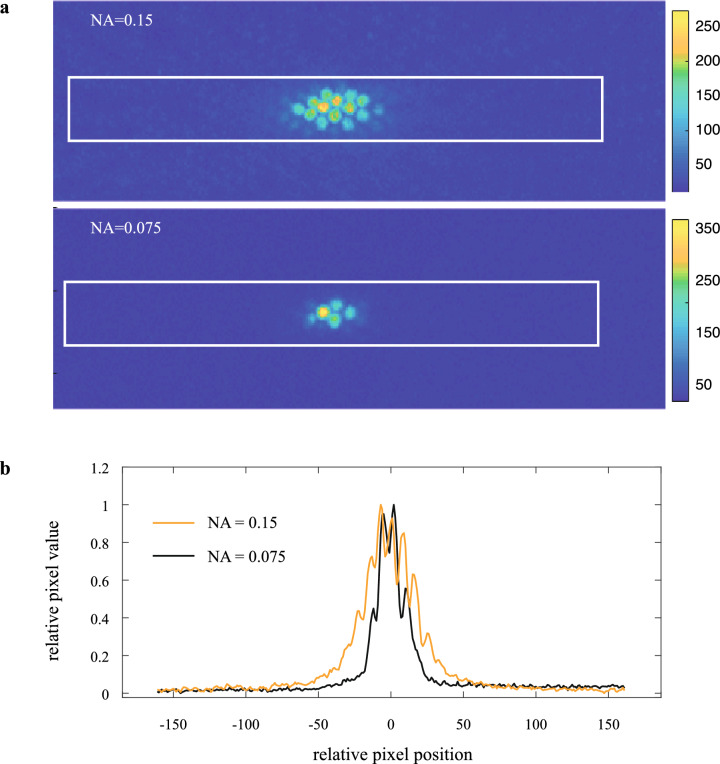
The dimensions of the induced fluorescent pseudopupil depends on the numerical aperture (NA) of the objective lens. **(a)** Heatmap of the induced fluorescent pseudopupil in a honeybee forager eye stained with Lucifer Yellow (see “Methods”) and acquired with a Nikon SMZ18 microscope fitted with a P2-EFLGFP-B filter cube, a 1 × SHR PlanApo objective with two different numerical apertures (NA), 0.15 (*top*) and 0.075 (*bottom*). **(b)** Relative pixel values of the *rectangular area* in **(a)** showing that the two pseudopupils have the same centre despite subtending a broader area when the NA is higher (*orange line*).

The narrower intensity profile of the induced fluorescent pseudopupil presumably reflects the double-pass nature of epifluorescence, where the resulting image depends both on excitation light being focused onto the fluorophores from the viewpoint of the observer, while off-axis light ends up absorbed by surrounding pigment cells without exciting fluorescence (Fig. 1b). Emission is then collected as for the luminous pseudopupil formed by antidromic illumination^22^. By contrast, the principal pseudopupil is dark over a langer area due to the same off-axis absorption of orthodromic light, but this time expanding the visible dark patch rather than exciting fluorescence (Fig. 1a). As also noted previously for luminous pseudopupils induced by antidromic illumination^22,29^, the width of the fluorescent area can be adjusted to a certain degree by the experimenter by varying the numerical aperture (NA) of the lens, either via switching objectives or by closing the iris diaphragm (Fig. 4), although this is a trade-off, due to decreased resolving power.

The small fluorescent pseudopupil allows easier determination of its centre compared to the dark pseudopupil, which can otherwise be problematic in determining angular sampling resolution^22^. Often, very broad pseudopupils are seen in species with intense acute zones where large numbers of photoreceptors look in almost the same direction (e.g. in dragonflies:^33^). These broad acute-zone pseudopupils are rarely circularly symmetric due to differences in the rate at which acuity falls off in different directions away from the centre. This can make it very difficult to determine the pseudopupil centre. For example, *A. cyanea* has a very broad, dark pseudopupil in the frontal part of the eye (Fig. 1c). However the induced fluorescent pseudopupil shows a much more easily identifiable centre (Fig. 1c and Supplementary Information video 1, part 1).

### (c) Eye maps for the honeybee forager compound eye confirm higher sampling resolution that prior estimates

The position of the pseudopupil can be used to determine Δ*φ* and thus the local sampling resolution of the eye^22,34^. This method involves estimating the shift in the pseudopupil centre (i.e. number of ommatidia that it moves across) for a known rotation of the eye. This has been used in a number of species, to obtain full maps of the change in Δ*φ* across the field of view of the eye, and thus provide valuable and unique information on how compound eyes sample the environment (e.g. in halictid and xylocopid bees:^35–37^).

**Figure 5:**
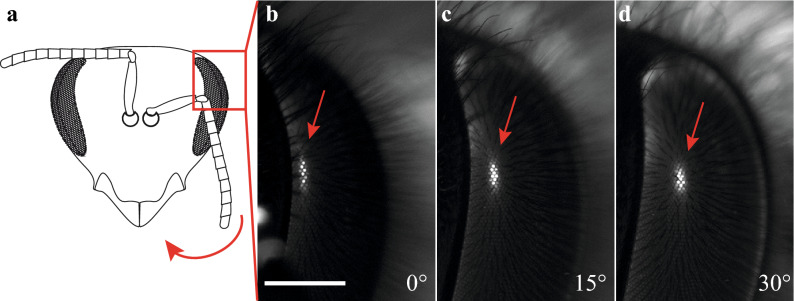
A induced fluorescent pseudopupil in the dark-pigmented compound eye of the honeybee, *Apis mellifera*. **(a)** The pigmented compound eye of a naive honeybee has no visible pseudopupil due to the presence of pigments around the retinula cells^61^. The *red arrow* shows the direction of rotation in **(b–d)**. **(b–d)** Zoomed-in view of the left eye of a honeybee forager, *Apis mellifera* (area of the eye as in the *red inset* in **(a)**), acquired using a Nikon SMZ18. The eye was stained with Lucifer Yellow as described in the Methods and the bright induced fluorescent pseudopupil is thus visible (*red arrow*) and shifts its location in the eye as the head of the honeybee is turned **(c, d)**. **(b)** The head is in its frontal view and rotated around its vertical axis at 15° and 30° in **(c, d)**, respectively. Scale bar: 500 µm.

**Figure 6:**
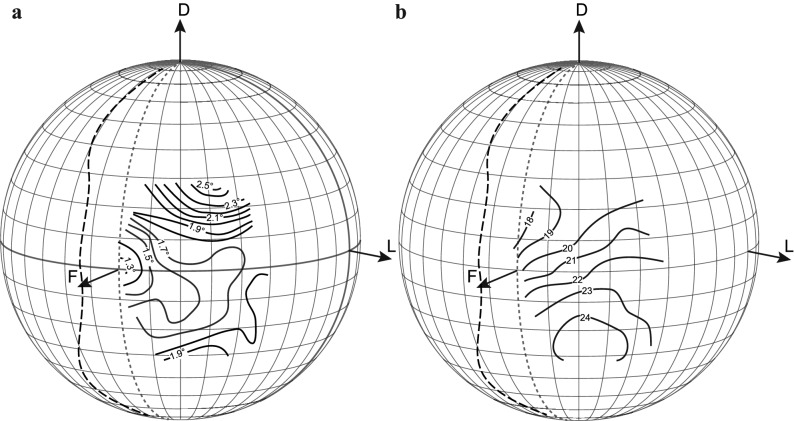
The induced fluorescent pseudopupil is a good tool for estimating the sampling resolution of a compound eye. **(a)** Projections of the interommatidial angle, Δ*φ*, (average of horizontal and vertical components) and **(b)** facet diameter (values in µm) of a single forager honeybee, *Apis mellifera*. The highest resolution is found in the frontal part of the eye where there is an averaged Δ*φ* of 1.3°. Note that the location of this minimum value does not coincide with the location in the eye having the largest facet diameter. The boundary of the visual field (*dashed lines*) is reconstructed from^38^. Dotted lines show 0° azimuth. *F* frontal, *D* dorsal, *L* lateral.

Given the sparseness of data (and past disagreements) on the sampling resolution of the honeybee forager due to the impossibility of visualising the dark principal pseudopupil in their heavily pigmented eyes, we selected this species as an ideal test for the practicality of our induced fluorescent pseudopupil method to map part of the eye (see Fig. 5 and Supplementary Information video 1, part 3). We rotated the head of a honeybee forager on a goniometer in 10° steps to scan a portion of the eye from −40° to + 40° in latitude and from −10° to 60° in longitude. The averaged 3D contour maps of Δ*φ* obtained (Fig. 6) revealed a frontal acute zone with a substantially higher resolution (i.e. lower Δ*φ*) across the frontal eye region than was previously described using the antidromic pseudopupil, anatomical or behavioural methods^6,16,26–30^. Along the equator, Δ*φ *stays below 1.7° to at least 40° of azimuth. Along the vertical orientation, resolution falls off more rapidly as one moves into the dorsal visual field, with frontal values of Δ*φ* already exceeding 2° by 20° of elevation. Resolution below the equator is more impressive, with Δ*φ *remaining below 1.9° down to −30° across the entire frontal region of the visual world. The centre of this acute zone corresponds to the frontal head axis, with a minimum Δ*φ* of just below 1.3°. Interestingly, this corresponds well with the region of the eye where we previously obtained the smallest acceptance angles, around 1.6°, using electrophysiological recordings^31^. Note that according to earlier maps of the visual fields^38^, this is a region of the eye with roughly 10° of binocular overlap (shown as a dashed line in Fig. 6). Hence, at the very front of the visual fields, objects viewed by the area of maximal acuity (Δ*φ * < 1.5°) would be seen by both eyes.

## Discussion

We used fluorescent dyes (Lucifer Yellow or Neurobiotin 488) to retrogradely stain the photoreceptors in compound eyes of living, intact, insects. We successfully obtained a clear, bright, pseudopupil that is visible both in clear compound eyes (*Aeshna cyanea, Eristalis tenax, Anthophora *sp.) and in dark, pigmented, compound eyes (*Apis mellifera*). Our data support the potential of this method for reliable pseudopupil analysis in a wide variety of compound eyes that have previously been problematic.

Although this method allowed us to obtain excellent pseudopupils, we observed a high variation in performance among species used and among samples. In particular, species with less pigmented eyes such as *Eristalis tenax* show the fastest uptake, with an induced fluorescent pseudopupil visible within 30 min, if kept in the dark. In dark, heavily pigmented eyes like those of *Apis mellifera,* the fluorescent pseudopupil took up to 12 h to be visible. Besides the presence of pigments, other factors may influence the penetration and uptake of the dyes, so future experimenters will need to optimise the dissection and application of the fluorophore for their selected target species. For example, we noticed differences in the rate of uptake depending on whether glands and tracheal tubes were removed, as well as how much the sample was subsequently exposed to light.

It has previously been reported that light might facilitate and speed up the endocytotic uptake of Lucifer Yellow by photoreceptors when injected through the retina^39^. We certainly observed a large increase in brightness of the eye during exposure to light while imaging under the epifluorescence microscope. Thus, in order to acquire images and map the pseudopupil across large areas of the eye it is important to minimize the exposure time, and to minimize the light intensity of both the microscope and the laboratory environment. Note that the dye diffuses throughout the haemolymph and other structures in the eye will also appear fluorescent (e.g. antennal pedicel and ocelli).

As we mentioned earlier, if left incubating for a long enough time, even in the dark (from about 3–4 h in the dark at RT for the drone fly *Eristalis*), the entire eye will end up glowing (Fig. 2e), frustrating further attempts to image the pseudopupil. We observed this for both Lucifer Yellow and for Neurobiotin 488. In future it may be worth additional experimentation with other neuronal tracers or fluorophores to see if these provide better control of this non-specific uptake.

While the light-driven uptake by other eye structures limits the time during which the induced fluorescent pseudopupil can be used to map visual acuity, the resulting fluorescence of a fully fluorescent eye may also be useful for other applications. For example, by constructing confocal image stacks of the corneal surface, we were able to obtain a complete 3-dimensional model of the corneal facets (Fig. 2e and Supplementary Information video 1, part 2). Accurate models of the whole corneal surface of the eye (and to a limited extent, of sub-corneal structures) could thus be obtained with a relatively simple procedure from a living eye, without fixing the head, similar to the structural illumination microscopy method used in non-fixed eyes. In this respect, this method may be superior for counting facet numbers, measuring facet diameters or for mapping corneal axes, compared with alternatives such as µCT imaging of fixed tissue^18,19^, or construction of facet maps from replicas made using nail polish that is peeled off the corneal surface^40–42^.

The method we present here establishes a basis for future comparative studies aimed at estimating the sampling resolution of dark, pigmented, compound eyes in intact animals. Compared to other techniques that physiologically record the sampling power of the eye (e.g. pattern electroretinogram^14,15^), the advantage of having a functional pseudopupil in a living eye is that it provides the easiest and most accurate method for mapping variations in visual acuity across the entire compound eye. Since many compound eyes show large variations in acuity across the eye, with specific acute zones associated with distinct differences in behaviour^1^, creating such maps is important for studying visual ecology and the evolution of compound eyes. Previous attempts to create such maps in dark-pigmented eyes have used antidromic illumination^7,17,30,43^ where light is passed through the back of the head of the insect. However, this preparation is often ex vivo and the head is prone to desiccation and thus to possible artefacts coming from misalignments between the light path and the ommatidial axis. Alternatively, in fixed compound eyes the ommatidial axis can be estimated anatomically. However, even if fixation is perfect, anatomical estimates are limited by departures from basic assumptions based on ideal eye geometry. The gaze direction for a single ommatidium is determined by the specific position of the tips of the receptors which lay together in different numbers and patterns depending on the type of compound eye and species (see for a review^44^). In an ‘ideal’ eye, each rhabdom is oriented such that its tip is co-axial with the overlying crystalline cone and the corneal lens. The angle between the receptor axes in two adjacent ommatidia (Δ*φ*) then provides a measure of the sampling frequency for that particular area of the eye. However, the actual axis of the rhabdom does not always coincide with the axis estimated from a simple anatomical reconstruction of the cornea and lenses (e.g. an axis perpendicular to the corneal surface). The latter, obtained from a fixed and sectioned eye, can deviate significantly from the receptor axis, particularly away from the centre of the field of view and in eyes with large local variations in acuity^22,45^. This was elegantly illustrated by Stavenga (Fig. 23 in^22^) for a damselfly, where the centre of the corneal reflection obtained by coaxial illumination of the eye rarely coincides with that of the dark pseudopupil. This problem is further exacerbated in sectioned material by possible distortions due to compression at the microtome knife edge.

As an alternative to histological sectioning, detailed morphological reconstruction of whole fixed eyes with X-ray micro computed tomography techniques (µCT) have recently been used in an attempt to map ommatidial axes in a number of species^46^. However, as with anatomical methods, only highly detailed maps of inter-facet angles can be made with this method. In order to overcome this limitation, Bagheri and colleagues^19^ used µCT but developed semi-automatic software to plot the rhabdom axis rather than the facet axis. Although these techniques are leading edge technologies for samples that cannot be kept alive in the lab or that are only available as museum specimens, or even fossils (e.g.^47^), they are complicated by the same assumptions as other anatomical methods. We suggest that our fluorescent pseudopupil provides a more direct and reliable result under normal physiological conditions.

Our application of this technique to the honeybee forager (*Apis mellifera*) resolves disparities between numerous past studies that have rarely used anything like a direct measure of the Δ*φ* (for a review see^15^). Both Kirshfeld^29^ and later Seidl^30^ used the antidromic illumination technique to visualize luminous pseudopupils in honeybees and then estimated a minimum Δ*φ* in the frontal part of the eye of 1.8° and 1.65° respectively (average of horizontal and vertical values). We found a similar minimum in a broad region in the frontal part of the eye, particularly in the ventral visual field, but were also able to map the centre of this acute zone, with Δ*φ* below 1.3°. This confirms that the frontal part of the eye has a much better resolution than previously estimated (Fig. 6). Recently, Taylor and colleagues^18^ used µCT to map the inter-facet angles of the eyes, as a proxy for estimating Δ*φ*. They found frontal inter-facet angles of around 1.6°, approximately 20% larger than we report here. However, this was not the area of maximum acuity that they report. They found a region with inter-facet angles down to 1° (i.e. even smaller than we report for the frontal eye fields), but this region was centred 60° more laterally, and 30° more dorsally than the directly frontal acute zone we have identified. A similar minimum was also found by Baumgärtner^16^, again 60° away from the anterior axis of the eye, but around the equator. However, as Taylor and colleagues do acknowledge, these techniques have limitations that arise from skewed rhabdom orientations. Any such skewness is, of course, fully accounted for when directly imaging the pseudopupil. Hence, we assert that our map definitively identifies the area of maximum acuity to be in the frontal visual field, in agreement with our recent electrophysiological determinations of photoreceptor acuity measured across the eye^31^. The location of this acute zone makes perfect sense considering that honeybees frontally fixate objects of interest, such as the hive entrance or flowers. It is also an eye region that is clearly involved in frontal pattern discrimination (e.g.^6,48,49^).

## Methods

### (a) Animals

Insects (*Apis mellifera*, *Eristalis tenax, Aeshna cyanea* and *Anthophora sp*.) were collected in the field between May and November 2019, anaesthetised on ice for a few minutes and then immobilized in a pipette tip cut at its narrow end using hot beeswax and violin rosin (1:1). The head was tilted forwards by 45° in order to gain access to the back of the head and the mouthparts were fixed with wax. We then visualised the pseudopupil in these species using both the traditional pseudopupil method and our novel fluorescence method.

### (b) The traditional pseudopupil method

This method has allowed reliable estimates of Δ*φ*, and even mapping of this angle over the entire eye, for a large range of species (see below). The method relies on the fact that a prominent dark pseudopupil is visible on the cornea when incident light from the gaze direction of the observer (e.g. for an eye viewed through a microscope or camera) is either absorbed or reflected by structures within the ommatidia.

In many insects, such axial incident light is absorbed by pigment cells surrounding the crystalline cone and the rhabdomeres of ommatidia directed towards the viewer and the corneal facet lenses of these ommatidia thus appear darker than those of more distant ommatidia receiving light from other directions^22,45^ (Fig. 1a). Since each photoreceptor samples an area of space that overlaps with its neighbouring ommatidia, this dark area extends across a small region of nearby ommatidia, giving the appearance of a dark ‘pupil’, by analogy to that in a camera eye. Unlike a real pupil however, this “pseudopupil” moves as the eye is rotated in front of a fixed viewpoint. As mentioned above, by measuring the shift in the centre of the pseudopupil for known small rotations of the eye, it is thus possible to map the ommatidial axes (and thus Δ*φ*) across the entire eye and to correlate this with changes in ommatidial facet diameter. Dark principal pseudopupils are seen in the apposition compound eyes of many species of insects and crustaceans (and have been exploited to measure Δ*φ*, e.g. in mantids:^45,50^; dragonflies:^33^; carpenter bees:^36,37^; hornets:^51^; and fiddler crabs:^52^).

In some species, the pseudopupil is not dark (as a result of light absorption) but is instead bright under coaxial illumination due to reflection of incident light from structures within the ommatidia, such as a tapetum lining the back of the eye beneath the rhabdoms (as in butterflies:^53,54^) or due to the presence of other reflective structures within the ommatidia such as screening pigments (e.g. water striders:^55^; flies:^22^; nocturnal bees:^35^). Just as with their darker counterparts, these luminous pseudopupils have proved particularly useful for mapping Δ*φ* in apposition eyes (e.g. butterflies:^34,56^; flies:^57–60^; water striders:^55^; nocturnal bees:^35^).

While the pseudopupil method provides a ‘gold standard’ technique for directly measuring sampling resolution in many species, its application to others is made difficult by the presence of large amounts of dark light-absorbing screening pigments below the cornea. This makes it difficult or impossible to distinguish the pseudopupil, unless antidromic illumination is used, i.e. by shining strong light through the head of the animal^7,29,38^, an approach which brings with it a suite of other problems.

### (c) A novel, fluorescence-based pseudopupil method

To visualise the fluorescent pseudopupil, the cuticle on the back of one eye was cut using a scalpel and set to one side. Glands (if present) and tracheal tubes were also gently removed. The haemolymph was partially removed with tissue paper and about 20 µl of a freshly made mixture of 1:1 Dimethylsulfoxide (Sigma-Aldrich Sweden AB) and Ringer’s solution was applied on the open head capsule. The insect was then left in the dark for 20 min at RT. A few small crystals of a fluorescent dye (we used either Neurobiotin 488 (BioNordika Sweden AB) or Lucifer Yellow (Sigma-Aldrich Sweden AB)) were then applied on the open head capsule before the head capsule was resealed by replacing the removed section of cuticle and waxing it in place to avoid desiccation. The insect was then left to incubate in a box containing wet tissue paper for 15 min-1 h (*Eristalis tenax* and *Aeshna cyanea*) or up to 6–12 h (*Anthophora sp*. and *Apis mellifera*) and kept in the dark at RT, to allow infiltration of the fluorophore.

### (d) Imaging

After incubation, each insect was imaged using either epi-illumination (for the fluorescent pseudopupil) or by using incident light in those species where the dark pseudopupil was visible (*Anthophora* & *Aeshna*), in a fluorescence stereomicroscope (Nikon SMZ18, BergmanLabora AB, Sweden) equipped with a GFP-B filter cube and Plan Apochromatic objectives: either 0.5× (working distance, WD, 71 mm), 1× (WD 60 mm) or 2× (WD 20 mm). Successful fluorescence labelling was seen as groups of ommatidia whose receptors were directed towards the objective lens, and clearly visible as a fluorescent patch (Figs. 1, 5), which then moved across the surface of the eye as the sample was slowly rotated along azimuth and/or elevation (Supplementary Information, video 1, part 1 and 3).

In order to acquire the images necessary to reconstruct a map of Δ*φ* across the compound eye, single animals were put on a Leitz goniometer and similar methods as used by Rutowski and Warrant^34^ were then applied (these methods, in turn, were based on those developed by Land and Eckert^57^—see^34^ for a full description of the methods). Briefly, the insect was carefully positioned at the centre of the goniometer with the surface of the back of the head parallel to the stage of the microscope (i.e. horizontal) and the anterior centre of the head aligned with the zeros of both the latitude (elevation) and longitude (azimuth) axes of the goniometer. The goniometer allowed us to tilt the head around its centre in both latitude and longitude. Before starting to acquire images, small fluorescent crystals (Lucifer Yellow, Sigma-Aldrich Sweden AB) were sprinkled on the surface of the eye with the use of a fine paintbrush to provide visible landmarks for subsequent off-line analysis. Images were acquired after moving the goniometer along lines of both azimuth (from −10° to + 60°) and elevation (−40° to + 40°), in 10° steps.

Images were then captured using a Nikon SMZ18 fluorescence stereomicroscope (Nikon, BergmanLabora AB, Sweden) that had been modified by a 180° reversal of the imaging head and rotation of the objective turret to align the episcopic light source (Sola light engine SM-5-LCR-SB Lumencor^®^, USA) coaxially with the imaging pathway of a cooled sCMOS camera (Andor Zyla 5.5, Oxford Instruments) coupled to NIS Elements AR software (version 4.50, Nikon, BergmanLabora AB, Sweden).

In order to acquire Fig. 1c and 5 and video 1 (part 1 and 3), animals were mounted in a custom-built goniometer constructed from 2 motorized precision rotation stages (KPRMTE/M, Thorlabs Inc., USA) mounted on a manual translation stage (all components from Thorlabs Inc., USA). This allowed continuous or stepwise rotation of the sample both along azimuth and elevation. To acquire Fig. 2 and video 1 (part 2), images were acquired with a Leica SP8 DLS confocal microscope (Leica Microsystems A/S, Denmark).

### (e) Analysis of eye maps

For each image captured after rotating the goniometer, the centre of the pseudopupil was obtained in facet row-based coordinates (see^33^), using the fluorescent landmarks on the eye. The average pseudopupil diameter at each location was measured by manually drawing a straight line through 5–8 ommatidia in focus near the centre of the pseudopupil using the Nikon software tool (NIS-Elements AR, version 4.50, Nikon, BergmanLabora AB, Sweden). A text file recording the centre of the pseudopupil (in facet row-based coordinates) for each combination of latitude and longitude, together with averaged facet diameter at the same location, were then fed into custom-built software (Facet version 4.0.0 for Mac OSX 2017,^33^) that allowed us to calculate the local average Δ*φ* (within a rhomboidal eye surface area 5 facets long and 5 facets wide along the two facet rows defining the coordinate axes). The software then generated 3D projection plots (representing the 3D space around the eye) with the calculated Δ*φ* and facet diameter given at each latitude and longitude. From these, contour maps showing changes in Δ*φ* and facet diameter within the visual field of the eye could be visualised as 3D projection plots. Note that the Δ*φ* obtained by this method represents the average of the angles subtended by nearest neighbouring ommatidia along all 3 hexagonally oriented rows of the eye, as used in a number of earlier studies^33–36,54,55,58^.

## Supplementary Information

Supplementary Video 1.

Supplementary Legends.

## Data Availability

The software Facet version 4.0.1 for Mac OSX 2017 is available from https://github.com/insectvision/Facet. Raw image data for Figure 6 are available on 10.5281/zenodo.5570599.
