# Publisher Correction: A new, fluorescence-based method for visualizing the pseudopupil and assessing optical acuity in the dark compound eyes of honeybees and other insects

**DOI:** 10.1038/s41598-021-01737-x

**Published:** 2021-11-09

**Authors:** Elisa Rigosi, Eric J. Warrant, David C. O’Carroll

**Affiliations:** Department of Biology, Lund University, Sölvegatan 35, 22362 Lund, Sweden

Correction to: *Scientific Reports* 10.1038/s41598-021-00407-2, published online 28 October 2021

In the original version of this Article a previous rendition of Figure 2 was published.

The original Figure 2 and accompanying legend appear below.

**Figure 2:**
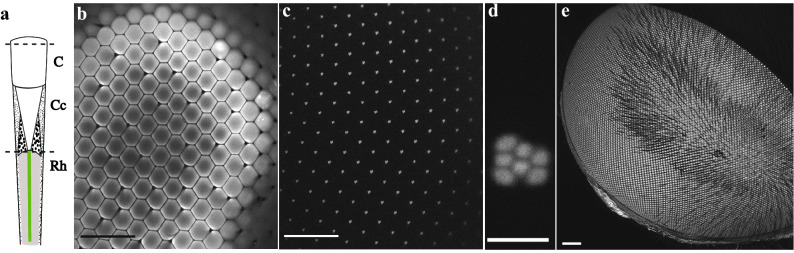
The fluorescence of the induced fluorescent pseudopupil originates from the rhabdomeres. **(a)** Diagram of a single ommatidium of a dipteran compound eye. Dotted lines denote the plane where optical cross sections of the eye in **(b–d)** were taken. From top to bottom: corneal facet (C), pseudocone (PC) and distal tip of the rhabdomeres (Rh). **(b,c)** *Eristalis tenax* compound eye after application of Lucifer Yellow and scanned with a confocal microscope, with a 63× glycerol objective. Scale bar 100 µm. The fluorescence observed at the surface of the eye **(b)** originates from the fluorescent rhabdomere tips as no other cells or parts of the photoreceptors are fluorescent when we focus below the cornea to the plane of the rhabdom tips **(c)**. **(d)** Magnified view from a single ommatidium, showing the distinctive trapezoidal shape formed by the distal tips of 7 adjacent rhabdomeres, typical of dipteran flies. Scale bar 5 µm. **(e)** Maximum intensity projection of a z-stack of the left eye of a female *Eristalis tenax.* When the dye (in this case Neurobiotin 488) had been left in the head for more than 3 h we experienced glowing in the entire eye. Image acquired with a Leica SP8 DLS confocal microscope and a 2.5× air objective lens (see also Supplementary Information video 1, part 2).

The original Article has been corrected.

