# Supplementary material for: A new, fluorescence-based method for visualizing the pseudopupil and assessing optical acuity in the dark compound eyes of honeybees and other insects
Source: Sci Rep. 2021 Oct 28;11:21267. doi: 10.1038/s41598-021-00407-2 (PMC8553845; doi:10.1038/s41598-021-00407-2)
Supplement: Supplementary file 2 — Supplementary Legends. [file 41598_2021_407_MOESM2_ESM.docx]

**Supplementary Information**

**Video 1**

**Part 1**

*Aeshna cyanea* stained with Lucifer Yellow (see Methods). Images acquired with a Nikon SMZ18 fluorescence stereomicroscope and with the animal mounted on a motorized precision rotation stage. Scale bar 500 μm, velocity 3°/s.

**Part 2**

Shows rotation of a volume-rendered projection of the 3D image stack obtained by confocal scanning laser microscopy for a fluorescent-labelled eye in a female hoverfly (*E. tenax*).

**Part 3**

Honeybee forager stained with Lucifer Yellow (see Methods). Images acquired with a Nikon SMZ18 fluorescence stereomicroscope from a honeybee mounted on a motorized precision rotation stage. Scale bar 500 μm, velocity 3°/s.
